# PuTmiR: A database for extracting neighboring transcription factors of human microRNAs

**DOI:** 10.1186/1471-2105-11-190

**Published:** 2010-04-15

**Authors:** Sanghamitra Bandyopadhyay, Malay Bhattacharyya

**Affiliations:** 1 Machine Intelligence Unit, Indian Statistical Institute, 203 B. T. Road, Kolkata - 700108, India

## Abstract

**Background:**

Some of the recent investigations in systems biology have revealed the existence of a complex regulatory network between genes, microRNAs (miRNAs) and transcription factors (TFs). In this paper, we focus on TF to miRNA regulation and provide a novel interface for extracting the list of putative TFs for human miRNAs. A putative TF of an miRNA is considered here as those binding within the close genomic locality of that miRNA with respect to its starting or ending base pair on the chromosome. Recent studies suggest that these putative TFs are possible regulators of those miRNAs.

**Description:**

The interface is built around two datasets that consist of the exhaustive lists of putative TFs binding respectively in the 10 kb upstream region (USR) and downstream region (DSR) of human miRNAs. A web server, named as PuTmiR, is designed. It provides an option for extracting the putative TFs for human miRNAs, as per the requirement of a user, based on genomic locality, i.e., any upstream or downstream region of interest less than 10 kb. The degree distributions of the number of putative TFs and miRNAs against each other for the 10 kb USR and DSR are analyzed from the data and they explore some interesting results. We also report about the finding of a significant regulatory activity of the YY1 protein over a set of oncomiRNAs related to the colon cancer.

**Conclusion:**

The interface provided by the PuTmiR web server provides an important resource for analyzing the direct and indirect regulation of human miRNAs. While it is already an established fact that miRNAs are regulated by TFs binding to their USR, this database might possibly help to study whether an miRNA can also be regulated by the TFs binding to their DSR.

## Background

MicroRNAs (miRNAs) are non-coding small RNAs (21-23 nt), which regulate mRNA stability and translation through the action of an RNA-induced silencing complex (RISC) [1,2]. Various biological processes, e.g., insulin secretion, cell proliferation, brain development, apoptosis etc., are controlled by miRNAs and emerging evidences strengthen the belief about their involvement in various diseases like fragile X syndrome, schizophrenia, cancer and many others [3-5]. Recent studies in systems biology suggest that there exists a complex regulatory network between genes, transcription factors (TFs) and miRNAs [6]. Study of such regulatory network is indeed promising for disease analysis in various organisms. The initiatives for exploring this comprehensive regulatory network primarily focus on pairwise regulation (e.g., gene-TF, gene-miRNA and TF-miRNA regulations) [6,7]. However, these approaches face a major problem due to the unavailability of proper repositories, specially for the regulating factors for miRNAs. In this paper, we focus on TF to miRNA regulation and provide a novel interface for extracting the list of putative TFs for human miRNAs. In the subsequent discussions, we use the representation TF→miRNA to denote a pair of TF and miRNA in which the TF might be a possible regulator of the miRNA.

There are very few earlier studies focusing on TF→miRNA relationships for a specific organism. Instead of being based on computational approaches, most of these rely on literature survey. In a recently published database, named TransmiR (version 1.0), a sum of 288 TF→miRNA relationships (of these 242 are unique), for 10 organisms, have been accumulated based on a manual survey [8] of about 5000 reports collected from PubMed Central [9]. Of these 242 distinct pairs, 221 pairs report about the regulation of human miRNAs. The complete list of TF→miRNA pairs includes 82 unique TFs and 100 unique miRNAs. Of these, 88 miRNAs and 69 TFs are related to the human category. Along with the regulation information, it also provides the information on the involvement of the TF with tumor and the miRNA with tumor and disease, respectively. Certainly, this is a very small fraction of the possible comprehensive network. Therefore, instead of depending only on literature survey there is a strong requirement of further in-depth computational analysis.

Here, we provide the first repository of the set of putative TFs for any arbitrary human miRNA binding in the possible regulatory regions (within 10 kb USR and DSR, respectively) of that miRNA. We call it hereafter as the repository of putative transcription factor to miRNA regulation (PuTmiR). It's up-to-date interface processes a region-specific search upstream of a given miRNA to find out the possible regulating TFs. As an additional feature, the web server implementation also provides the putative TFs binding to the downstream region of miRNAs. In spite of the conventional focus on upstream regulatory factors of genes, PuTmiR also provides the data for downstream regions as this might possibly be useful in exploring the fact whether miRNAs could be regulated from downstream regions or not. This kind of interface is a novel one for the system level study of miRNA regulation.

## Contents and construction

The complete list of human (*Homo sapiens*) miRNAs, which have stem-loop structure precursor sequence validated with literature evidence, and their corresponding genomic locations have been collected from the up-to-date repository of miRBase [10]. This produces a list of total 721 miRNAs. Some of the miRNAs have been discarded from this list because they are found absent from the assembly of UCSC [11]. Notably, we have used UCSC to extract some additional information on TF binding sites. Specifically, several chromosomal entries like MT (mitochondrial) and HS chromosomes are found to be unusable for this study. The chromosomes HSCHR6_MHC_COX, HSCHR6_MHC_MANN, HSCHR6_MHC_DBB, HSCHR6_MHC_MCF, HSCHR6_MHC_QBL containing hsa-mir-219-1, HSCHR6_MHC_COX, HSCHR6_MHC_DBB, HSCHR6_MHC_MCF, HSCHR6_MHC_MANN, HSCHR6_MHC_QBL, HSCHR6_MHC_SSTO containing hsa-mir-877, HSCHR6_MHC_COX, HSCHR6_MHC_DBB, HSCHR6_MHC_MCF, HSCHR6_MHC_QBL, HSCHR6_MHC_SSTO containing hsa-mir-1236, and MT containing hsa-mir-1974, hsa-mir-1977 and hsa-mir-1978 as mentioned in the miRBase have been discarded. To search for the TFs we retrieved data from the UCSC genome browser [11]. We define the USR and DSR with respect to the 5' and 3' untranscribed end of an miRNA, respectively. Again, hsa-mir-941-4 has been removed from the list because its chromosomal location is empty in the miRBase repository.

After collecting the data on the genomic locations of the reduced set of miRNAs, we have searched for the transcription factors (TFs) binding to the 10 kb USR and DSR of the individual miRNAs. 10 kb is considered to be a standard region of interest of genes for regulatory analysis [6]. The entire assembly of putative TFs have been collected from the March 2006 assembly (up-to-date repository on binding TFs) of UCSC genome browser [11]. We have extracted the putative set of TFs binding to required regions of interest. The requisite table has been downloaded from the UCSC Table browser retriever tool [11] by setting the following options:

• clade: Mammal,

• genome: Human,

• assembly: Mar. 2006,

• group: Regulation,

• track: TFBS Conserved,

• table: tfbsConsSites and tfbsConsFactors.

While defining the USR or DSR, if the chromosomal boundaries are crossed, then the bounds are set to 1 or the maximum base pair location, as the case may be. However, the UCSC assembly on transcription factor binding sites (TFBSs) being a bit outdated (Mar. 2006), some of the existing entries are found to be inconsistent with the miRBase information. Some of the genomic locations collected from the miRBase database are not valid in the outdated repository of UCSC. So, these entries have been discarded. These miRNAs and their corresponding genomic locations are reported in Table 1.

**Table 1:** The list of genomic locations of miRNAs in miRBase that are invalid according to the Mar. 2006 repository of UCSC.

MiRNA name	Chromosome	Start location	End location	Strand
hsa-mir-338	17	79099683	79099749	-
hsa-mir-602	9	140732871	140732968	+
hsa-mir-647	20	62573984	62574079	-
hsa-mir-657	17	79099076	79099173	-
hsa-mir-941-1	20	62550794	62550882	+
hsa-mir-941-2	20	62551101	62551189	+
hsa-mir-941-3	20	62551213	62551301	+
hsa-mir-1250	17	79106996	79107108	-
hsa-mir-1302-2	15	102500662	102500799	-
hsa-mir-1914	20	62572818	62572897	-

The list of genomic locations of miRNAs collected from miRBase that are found to be invalid according to the Mar. 2006 repository of UCSC genome browser. The locations are given in base pairs (bp).

The UCSC uses the public version of the Transfac binding site distribution matrix (TRANSFAC^®^7.0) [12] for predicting putative TFs. The Transfac public repository contains position-weight matrices for 398 TFBSs characterized through empirical studies reported in the scientific literature. From this, 258 binding matrices for known TFs conserved in the human, mouse or rat alignment (i.e., the prediction accuracy is higher than a threshold for its binding matrix over all the species considered) were used in the tfbsConsSites track of UCSC. These matrices are used for predicting the TFBSs in a genomic location. Again, the tfbsConsFactors track contains the TFs corresponding to these transcription factor binding sites. By combining these two tracks, we have retrieved the list of putative TFs.

For predicting the TFBSs, the matching scores corresponding to the binding matrices are computed for each genomic location and for all the species. Here 3 species, human, mouse and rat are considered.

Suppose, the multiple species alignment is represented as *s*, where *s*_*ji *_denotes the nucleotide at position *j *of species *i*. Again, suppose *B *to be a matrix (of dimension 3 × 4) giving the background frequencies of each nucleotide in each species. A sliding frame of length *n *is used to compute the score (*ss*) for each species at each position *i *as(1)

From this, a log-score is calculated for each species (normalizing by the length of the matrix and the number of species in the alignment) as(2)

From this, a log-odds score for the current position is derived by adding up the scores computed in Eqn. (2) for all the species. The log-odds score of each species must exceed the threshold for that species. It is finally used for the prediction of TF binding sites. The prediction of putative TFs returns a z-score that denotes the significance of the prediction. For an entry *i *the z-score is computed as(3)

where *μ*_*log_score *_and *σ*_*log_score *_in Eqn. (3) denote the mean and standard deviation of the *log_score*s, respectively, derived for all the cases. This z-score is then used to create the threshold for each binding matrix, namely, 1.64 standard deviations above the mean. The threshold is calculated for each species such that the maximum *p*-value becomes 0.05. Tfloc is then run with this threshold on each chromosome for the 3-way multiz alignments.

### Preparing the final dataset

By combining the retrieved information, we have produced the final results in the form of two complete tables for 10 kb USR and DSR, respectively. Notably, the TFs extracted through these processes are taken from a table which contains the location and score of TFBSs conserved in the human/mouse/rat alignment. A conserved binding site across the alignment is defined for which the score is greater than or equal to a threshold for its binding matrix in all these three species. Therefore, the extracted TFs are specific to the organisms human, mouse and rat. As we are focusing on the human TF→miRNA relationships in this study, the TFs specific to mouse and rat are pruned out from the data. In this way, we have finally obtained at least one putative TF binding to the 10 kb USR of 601 miRNAs and in the 10 kb DSR of 613 miRNAs, respectively. In total, 11485 and 12251 relationships are found where a TF binds in the 10 kb USR and DSR of the human miRNAs considered in the study, respectively. In course of the preparation of the final dataset, We have included the Refseq IDs corresponding to the TFs considered in the list. The complete datasets for upstream region and downstream region are provided in Additional files 1 and 2, respectively.

**Figure 1:**
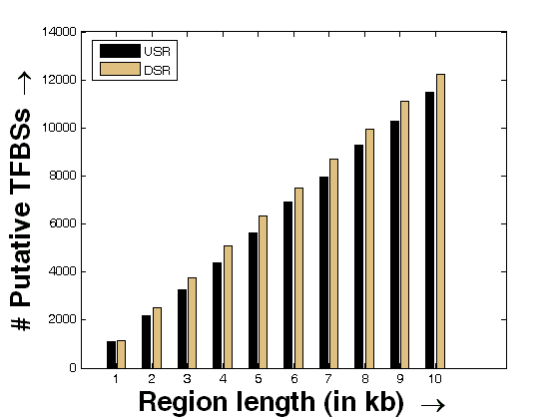
**Comparative cumulative distribution of the number of putative TFBSs in the discretized regions of USR and DSR**. Comparative cumulative distribution of the number of putative TFBSs (based on the list of TFs found to bind) in the USR and DSR divided into 10 discrete fragments of 1 kb each. The bars in black and metallic gold correspond to the number of total putative TFs binding in the USR and DSR, respectively within the region defined along the x-axis.

The comparative cumulative distributions of the binding sites of the TFs found within the upstream and downstream genomic locations of the miRNAs are shown in Figure 1. The 10 kb USR and DSR have been separated into 10 discrete regions (1 kb each) along the x-axis. For each of this region, the number of total TFs that binds within this region are shown in bars for USR and DSR, respectively. As it is evident from the figure, the number of TFs cumulatively grows more in the plot for the case of DSR as compared to the case of USR after the 1 kb region. This is an interesting observation as this might indicate that the miRNAs could possibly be regulated from their downstream regions.

### Analyzing the data

**Figure 2:**
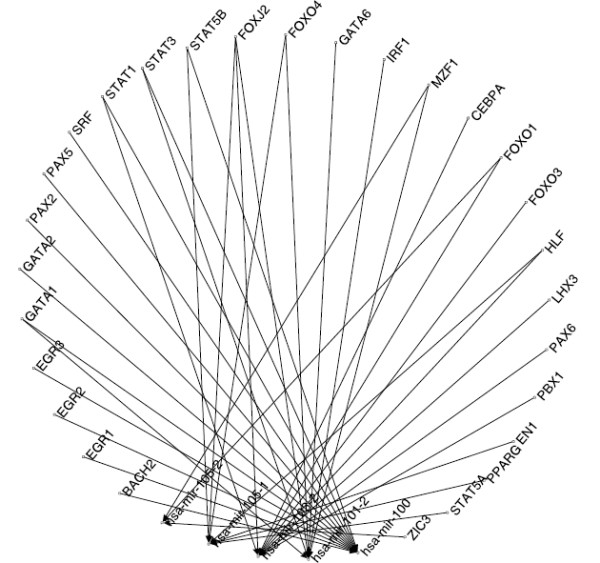
**A partial view of the entire bipartite graph obtained between the TFs and the miRNAs studied**. A portion of the total directed graph, in the form of a bipartite, constructed with the regulatory relations obtained. It shows regulatory interactions from 28 putative TFs towards 5 miRNAs.

**Figure 3:**
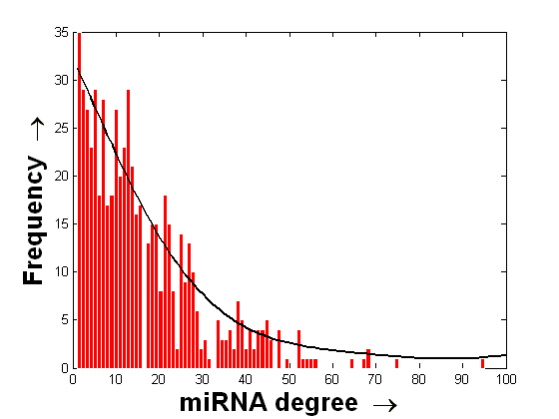
**Histogram of the degree values of miRNAs with respect to the TFs binding to their 10 kb USR**. Histogram of the degree values of miRNAs with respect to the number of unique putative TFs binding to their 10 kb upstream region. The histogram is shown using the red bars. The distributed values along the x-axis are finally fitted with a curve that is shown in black.

**Figure 4:**
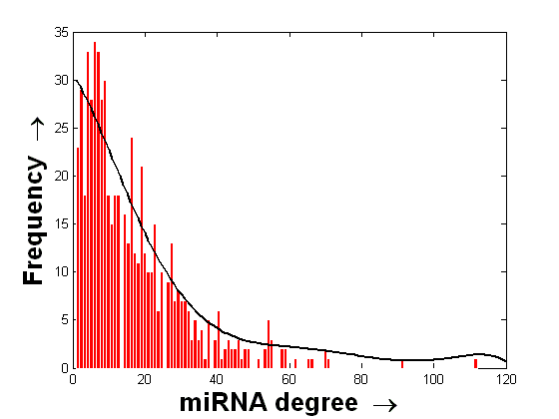
**Histogram of the degree values of miRNAs with respect to the TFs binding to their 10 kb DSR**. Histogram of the degree values of miRNAs with respect to the number of unique putative TFs binding to their 10 kb downstream region. The histogram is shown using the red bars. The distributed values along the x-axis are finally fitted with a curve that is shown in black.

It may be noted that several TFs are found to have more than one binding region within the 10 kb USR and DSR of an miRNA. We now extract out the distinct TF→miRNA pairs from the obtained list. This results in a total of 9606 and 10122 unique TF→miRNA regulatory pairs in the 10 kb USR and DSR, respectively. Let us assume that the network of regulatory relations found between the TFs and human miRNAs form a bipartite graph . Here,  denotes the set of TFs,  denotes the set of human miRNAs and  denotes the regulatory interactions obtained earlier. A partial view of the bipartite graph G for the case of 10 kb USR, comprising 5 miRNAs and 28 TFs, is shown in Figure 2. The regulatory structure of the entire graph can be realized from this partial view. The histogram of the degree values of the miRNAs in the entire graphs for the cases of USR and DSR, respectively, are shown in the Figures 3 and 4. The mean and standard deviations of these two distributions are ⟨15.98, 13.45⟩ and ⟨6.51, 14.4⟩ for the USR and DSR, respectively. As expected, these two distributions roughly follow a power-law nature. It can be seen from these two figures that we have higher number of miRNAs that have at least one putative TF binding to their 10 kb DSR as compared to the case of 10 kb USR. Again, the maximum degree is found to be 112 for DSR as compared to 95 for USR.

**Figure 5:**
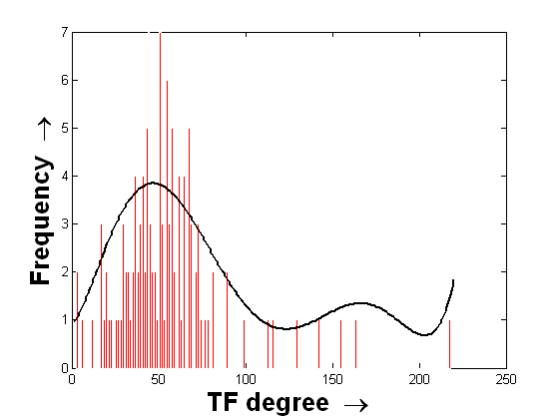
**Histogram of the degree values of TFs considering their binding to the 10 kb USR of miRNAs**. Histogram of the degree values of putative TFs considering the number of unique miRNAs to which they bind within the 10 kb upstream region. The histogram is shown using the red bars. The distributed values along the x-axis are finally fitted with a curve that is shown in black.

**Figure 6:**
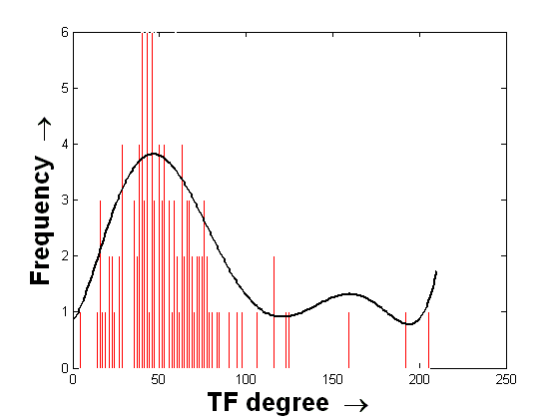
**Histogram of the degree values of TFs considering their binding to the 10 kb DSR of miRNAs**. Histogram of the degree values of putative TFs considering the number of unique miRNAs to which they bind within the 10 kb downstream region. The histogram is shown using the red bars. The distributed values along the x-axis are finally fitted with a curve that is shown in black.

The histograms of the degree values of the TFs are shown in Figures 5 and 6. The mean and standard deviations of these two distributions are ⟨54.27, 29.37⟩ and ⟨57.51, 30.85⟩ for the USR and DSR, respectively. Interestingly, these two histograms show that the frequencies of the TF degrees attain their maximum values towards the means of the distributions. In other words, the number of putative TFs that bind in the possible regulating region of a very small or a very large number of miRNAs is less. Biologically, it can be stated that most of the miRNAs are regulated by single TFs while many TFs regulate on an average around 50 miRNAs. Here, the frequencies of the TF degree values are found to be slightly higher for the case of USR than the case of DSR. This information raises the scenario of the possible regulation of miRNAs from both the 5' and 3' end, which is an interesting hypothesis to investigate.

We have studied the significance of the TF→miRNA data through comprehensive literature survey. We have found a couple of TFBSs in chromosome 1, which corresponds to the binding site of the TF YY1, in the upstream of hsa-mir-29b-2 and hsa-mir-29c, and report it in the PuTmiR database. This information has earlier been accounted in a study that reveals the repression of hsa-mir-29b and hsa-mir-29c through YY1 protein [13]. It also validates the association of the regulation with lung cancer, Tcl1 expression, Mcl-1 protein expression and apoptosis. This is a significant support to the putative TFs found for the human miRNAs. Moreover, we have found a total of 114 miRNAs (and reports in PuTmiR) whose common putative TF is the YY1 protein. Based on the reports in a recent survey in [14], we identified that 13 of these miRNAs (hsa-let-7a-1, hsa-let-7e, hsa-let-7f-2, hsa-mir-107, hsa-mir-135b, hsa-mir-143, hsa-mir-145, hsa-mir-199a, hsa-mir-21, hsa-mir-218-2, hsa-mir-29b-2, hsa-mir-29c, hsa-mir-375) are related to colon cancer. Interestingly, the miRNAs hsa-mir-29b-2 and hsa-mir-29c, which are biologically validated targets of YY1 [13], also belongs to this list. So, most likely this group of miRNAs is regulated by the YY1 protein acting as the TF.

## Implementation

The PuTmiR web server has been designed for providing a custom interface for extracting TFs of human miRNAs for a specific binding region of interest. At the lower level, we have the complete list of putative TFs binding to the 10 kb USR and DSR of the miRNAs, where the binding confidence is based on the position-weight matrices stored in the TRANSFAC database [12]. We provide an optional interface to define the region for which the TFs are required to be extracted for upstream or downstream. The server side code of the interface is written in PHP and has been made non-specific to browsers. The extracted output in the tabular form provides the genomic locations, strand, locations of interest, location, name and strand of TFs, and z-score of the binding prediction accuracy for the miRNA given as query. The extracted data can also be downloaded in tabular format.

**Figure 7:**
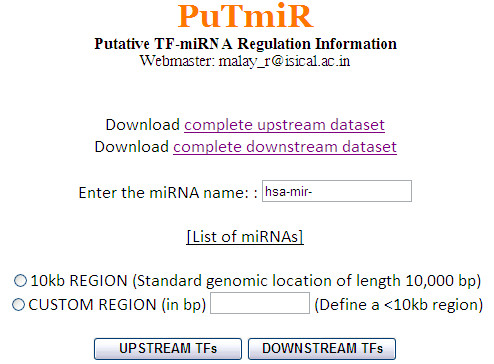
**A view of the front interface of the web server**. The front interface of the web server for extracting putative TFs binding in the user-defined upstream or downstream region of an miRNA.

A front view of the web server along with the optional interface is shown in Figure 7. The miRNA name is to be given as the query to search for the putative TFs. In addition to this, the region of interest is to be provided by selecting either 10 kb or any custom region (any positive range less than 10 kb) using radio buttons. If the custom region is selected, the user has to feed the region (in base pairs) using a text field.

The user-defined region is restricted not to exceed 10 kb because the 10 kb region is expected to be the possible regulating region of any miRNA gene [6,7]. Finally by clicking on the upstream or downstream optional buttons, the putative TFs are extracted. The results are available in tabular format or in downloadable text format. As an interesting feature of PuTmiR, we have included a link to the PubMed central database, which is regularly updated with published scientific literature, to browse for the regulation information (by TF) on the miRNA given as query.

**Figure 8:**
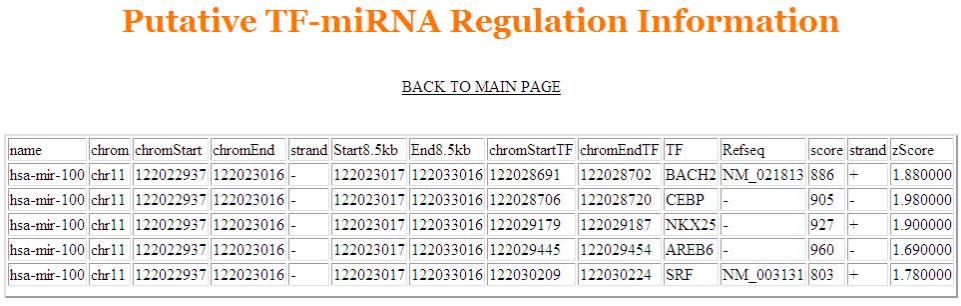
**A view of the extracted data returned by the web server**. The extracted data shown by the web server for the TFs binding in the 8.5 kb upstream region of the miRNA hsa-mir-100. The extracted data in tabular format shows the miRNA name along with the putative binding TFs and their binding regions.

A view of the output page (in tabular format), producing the extracted list of putative TFs through PuTmiR, is shown in Figure 8. The thirteen columns of the table represent the name, chromosome, starting location, ending location, strand, staring location of interest, ending location of interest, TF name, score of prediction of binding, TF strand and z-score of the binding prediction accuracy, respectively of the corresponding miRNA. Using the comprehensive integration procedure, the web interface is capable of providing not only a possible list putative regulators of human miRNAs but also their binding locations and strength.

## Usefulness of the data

The data evolving as an output from this study is important in the perspective of further system level analysis. Systems biology involves analysis in the lower molecular levels, which mean in the levels of genes and proteins (including TFs). Recent addition to this list is the miRNAs that actively participate in the negative regulation of genes [1], and thus proteins. It is conjectured that there exists regulatory interactions between genes, TFs and miRNAs [6]. Additionally, current studies strengthen the fact that while TFs regulate the miRNAs, miRNAs themselves target (directly or indirectly) signaling proteins, enzymes and TFs, and they all are believed to form a complex regulatory network [15]. Thus, the putative TF→miRNA data is useful in a systems biology context. It can be integrated and mapped onto various networks for various studies.

A handful of studies have been carried out in the recent past to integrate regulatory information with different biological networks. In one of these few, Cui *et al*. have mapped interactions between miRNAs to a human cellular signaling network for further regulatory analysis. They identified that miRNAs preferentially target the positive regulatory motifs, highly connected scaffolds and most downstream network components (e.g., signaling TFs). On the other hand, the negative regulatory motifs, common components of basic cellular machines and most upstream network components (such as ligands) are found as weak candidates to be a target [15]. Similar analysis has also been done with metabolic networks [16] and protein networks [17]. In [16], miRNA targets have been mapped onto the enzymes of a human metabolic network and statistically analyzed to discover novel regulatory patterns. They concluded that highly connected enzymes are potential targets of majority of the miRNAs. As the regulation of metabolic enzyme-encoding genes (by the miRNAs) controls the production of metabolites, the regulation of miRNAs is itself an important study to pursue. Similarly, miRNA regulation has also been studied over the protein-protein interaction networks [17]. The TFs which regulate miRNAs is therefore the key factors of indirect regulation of different biological networks.

Pursuing their earlier study in [15], Cui *et al*. have integrated cancer mutated and cancer-associated methylated genes onto a human cell signaling network to investigate the oncogene-signaling map [18]. They found that the genes in the downstream of the network are frequent targets for activating mutations. Most importantly, the genes highly connected to mutated genes are mostly cancer associated. Recent studies on the construction of disease regulatory networks, as published on specific diseases like cancer upon well-organized surveys [14], are already in progress. These studies can thus be further extended from the TF→miRNA regulatory information available in the database. One of our earlier studies shows that co-expressed miRNAs have common TFs, by analyzing a stem cell dataset [7]. Ongoing investigations have already revealed the involvement of miRNAs in stem-cell biology [19]. So, this type of data on miRNAs is indeed promising for exploring various disease regulatory activities of miRNAs, directly or indirectly.

## Biological insights into downstream regulation

In this work, alongside providing the upstream neighboring TFs of miRNAs in the PuTmiR web server, we also accumulate the TFs binding in the DSR of an miRNA. While analyzing the DSR data, we have identified some potential TF→miRNA pairs, i.e. some corresponding putative TFBSs within the 10 kb downstream region of some miRNAs, which have been biologically validated earlier. It is an established fact that the TF p53 upregulates hsa-mir-192 [20]. In our study, we have found four putative TFBSs of p53 within the 10 kb downstream region of the miRNA 192. On the other hand, for each of the proteins MEF2 and YY1, a single putative TFBS has been identified within the 10 kb DSR of the miRNAs 133a-1 and 29b-2, respectively, which are already known to be validated TFs of these miRNAs. Recent studies have revealed that MEF2 regulate the expression of a bicistronic miRNA cluster encoding hsa-mir-133a-1 in cardiac and skeletal muscle [21], and also, the derepression of hsa-mir-29b is caused by the downregulation of YY1 protein [13]. Therefore, some of these putative findings are also biologically convincing. It is an important recent concern whether the miRNAs possibly be regulated by the TFs binding to their downstream region. In fact, there is hardly any comprehensive study establishing this fact. However, we summarize the few in literature for reviewing current beliefs on downstream miRNA regulation. Earlier in 2002, *in silico cloning *was used in a study to identify a putative promoter region located about 215 bp downstream of the miR16 gene [22]. The investigations on conserved regulatory motifs in 3' untranscribed regions (UTRs) over multiples species were originally started by Xie and his colleagues [23]. They have identified 72 highly conserved (over the species human, mouse, rat and dog) 3' UTR 8-mer motifs which are most possibly binding sites of miRNA genes. In a recent biological analysis on the tumor suppressor PRDM5 (binds in 41% of the promoter sequences), it is found to occupy the proximal region (within the 1 kb upstream or downstream of mature miRNA sequence) of 10% miRNAs [24]. They identified the binding sites to be intronic, intergenic or within the exons of protein-coding genes. In another study on the roles of Myeloid transcription factors in the transcription of Pri-miR-223 in mouse, it is figured out that the mutation in the downstream PU.1-binding motif results in a drastic decrease of transcriptional activity [25]. It suggests a dominant role of the corresponding site in transcriptional control of mouse pri-miR-223. These results demonstrate that transcription of mouse pri-miR-223 is optimally induced in the presence of both of two PU.1-binding sites in the conserved promoter, therefore implying a critical role of PU.1 in pri-miR-223 transcription.

In a relatively recent analysis on the distribution of CpG islands, a useful aid for promoter prediction, Saini *et al*. have identified a good number of candidates at the downstream of intergenic miRNAs [26]. They strongly conjectured the existence of TF-binding sites in the downstream of miRNAs and also the presence of distal regulatory elements like enhancers or silencers regulating therein. The enhancers, a class of regulatory elements, can modify expression of a nearby gene when they are present, on either strand, upstream or downstream, and in either orientation. They can even be located relatively far from the gene regulated. Later, Saini and his group have studied many conserved primary transcripts of intergenic miRNAs as a follow through of their earlier work [27]. These studies indeed suggest that TF binding sites of miRNA genes might act as enhancers. Also, there are current evidences that proposes the initialization of miRNA transcripts in the upstream or downstream of promoter regions of miRNAs [28]. So, downstream regulation of miRNAs is in fact an interesting due study.

## Conclusions

This paper describes a novel repository of putative TF→miRNA information. This repository provides promising information for further study. The downstream regulatory activities of miRNAs, which is hitherto an unproved hypothesis, can be statistically studied using the information on putative TFs binding to the DSR of miRNAs provided by this study. It is indeed promising to analyze whether bi-directional regulation really happens for miRNAs or not. In fact, it is really surprising to receive a longer list of putative TFs binding in the DSR of miRNAs as compared to the USR. These observations may lead to further analyzes in this regard. It is an important concern in a study like this that miRNAs are of two types - intragenic and intergenic, according to their genomic locality. So, it may happen that the promoter of an miRNA is in the upstream of the gene from which it is encoded, without being in the exact upstream of the miRNA. Further studies pointing towards these directions are really important. It is a limitation that we are using the miRBase and UCSC genome browser at the intermediate stages of this analysis. The web server suffers from the incompatibility issues arising between these two repositories. This could be improved by retrieving updated TF information from Transfac to predict putative TFs more accurately compatible to genomic locations provided in miRBase. We plan to further improve the database by incorporating the literature-validated support in the regulatory relations obtained.

## Availability and requirements

The complete datasets of putative TFs for both the 10 kb USR and DSR are provided. The web server implementation can be accessed at the link: http://www.isical.ac.in/~bioinfo_miu/TF-miRNA/TF-miRNA.html.

## Authors' contributions

SB and MB jointly carried out the literature survey and pre-work planning, and conceived of the complete study. MB prepared the draft version of the manuscript. SB gave insightful suggestions for significant improvements. Both the authors read and approved the final version of the manuscript.

## Supplementary Material

Additional file 1 **List of putative TF→miRNA information on the 10 kb USR**. The list of putative TFs that have binding sites in the 10 kb upstream regions of the miRNAs. This tabular data includes the miRNA names, along with their genomic locations (chromosome name, strand, start site and end site), putative binding TFs and their genomic locations (strand, start site of binding and end site of binding) and strength of binding prediction (prediction score and z-score). Click here for file

Additional file 2 **List of putative TF→miRNA information on the 10 kb DSR**. The list of putative TFs that have binding sites in the 10 kb downstream regions of the miRNAs. This tabular data includes the miRNA names, along with their genomic locations (chromosome name, strand, start site and end site), putative binding TFs and their genomic locations (strand, start site of binding and end site of binding) and strength of binding prediction (prediction score and z-score). Click here for file
